# Quantification of perivascular inflammation does not provide incremental prognostic value over myocardial perfusion imaging and calcium scoring

**DOI:** 10.1007/s00259-020-05106-0

**Published:** 2020-11-16

**Authors:** Susan Bengs, Achi Haider, Geoffrey I. Warnock, Michael Fiechter, Yves Pargaetzi, Georgios Rampidis, Dominik Etter, Winandus J. Wijnen, Angela Portmann, Elena Osto, Valerie Treyer, Dominik C. Benz, Alexander Meisel, Tobias A. Fuchs, Christoph Gräni, Ronny R. Buechel, Philipp A. Kaufmann, Aju P. Pazhenkottil, Catherine Gebhard

**Affiliations:** 1 Department of Nuclear Medicine, University Hospital Zurich, 8091 Zurich, Switzerland; 2 Center for Molecular Cardiology, University of Zurich, 8952 Schlieren, Switzerland; 3 Swiss Paraplegic Center, 6207 Nottwil, Switzerland; 4 Institute of Clinical Chemistry, University of Zurich, 8091 Zurich, Switzerland; 5 University Heart Center, University Hospital Zurich, 8006 Zurich, Switzerland; 6 Institute for Regenerative Medicine, University of Zurich, 8952 Schlieren, Switzerland

**Keywords:** Fat attenuation index (FAI), Myocardial perfusion imaging (MPI), Coronary artery calcium scores (CACS), Gender bias

## Abstract

**Aims:**

Perivascular fat attenuation index (FAI) has emerged as a novel coronary computed tomography angiography (CCTA)–based biomarker predicting cardiovascular outcomes by capturing early coronary inflammation. It is currently unknown whether FAI adds prognostic value beyond that provided by single-photon emission computed tomography myocardial perfusion imaging (SPECT-MPI) and CCTA findings including coronary artery calcium scoring (CACS).

**Methods and results:**

A total of 492 patients (mean age 62.5 ± 10.8 years) underwent clinically indicated multimodality CCTA and electrocardiography (ECG)-gated ^99m^Tc-tetrofosmin SPECT-MPI between May 2005 and December 2008 at our institution, and follow-up data on major adverse cardiovascular events (MACE) was obtained for 314 patients. FAI was obtained from CCTA images and was measured around the right coronary artery (FAI[RCA]), the left anterior descending artery (FAI[LAD]), and the left main coronary artery (FAI[LMCA]). During a median follow-up of 2.7 years, FAI[RCA] > − 70.1 was associated with an increased rate of MACE (log rank *p* = 0.049), while no such association was seen for FAI[LAD] or FAI[LMCA] (*p* = NS). A multivariate Cox regression model accounting for cardiovascular risk factors, CCTA and SPECT-MPI findings identified FAI[RCA] as an independent predictor of MACE (HR 2.733, 95% CI: 1.220–6.123, *p* = 0.015). However, FAI[RCA] was no longer a significant predictor of MACE after adding CACS (*p* = 0.279). A first-order interaction term consisting of sex and FAI[RCA] was significant in both models (HR 2.119, 95% CI: 1.218–3.686, *p* = 0.008; and HR 2.071, 95% CI: 1.111–3.861, *p* = 0.022).

**Conclusion:**

FAI does not add incremental prognostic value beyond multimodality MPI/CCTA findings including CACS. The diagnostic value of FAI[RCA] is significantly biased by sex.

## Introduction

Recently, mapping of perivascular fat surrounding the coronary arteries was proposed as a tool for early non-invasive detection of coronary inflammation. Perivascular adipose tissue (PVAT) resides at the adventitial border, thereby modulating vascular homeostasis and regulating the local microenvironment via release of a wide range of bioactive molecules including adipocytokines [1, 2]. The adipogenesis of immature preadipocytes is promoted by peroxisome proliferator-activated receptor (PPAR)-γ activation; however, this mechanism is substantially inhibited by exogenous inflammation in the surrounding vasculature, which can be measured by routine coronary computed tomography angiography (CCTA). Accordingly, the perivascular fat attenuation index (FAI) was recently developed as a CCTA-derived imaging metric that can detect coronary inflammation and has demonstrated a prognostic value in the early detection of adverse cardiovascular events [2]. Indeed, it has been reported that perivascular FAI improves cardiac risk prediction over conventional CCTA prompting significant patient reclassification for cardiac and all-cause mortality [2]. However, it is currently unknown whether FAI quantification adds a prognostic value as an adjunct to nuclear myocardial perfusion imaging (MPI) and cardiac multimodality imaging comprising ^99m^Tc-tetrofosmine single-photon emission computed tomography (SPECT)-MPI and CCTA. Further, it remains elusive whether women and men benefit equally from FAI quantification. Hence, we assessed whether FAI adds incremental information gain for predicting major adverse cardiovascular events (MACE) beyond that provided by SPECT-MPI and CCTA findings in women and men undergoing cardiac multimodality imaging.

## Methods

### Study population

Our study population consists of 492 outpatients (173 women, 35%) who underwent clinically indicated CCTA and ^99m^Tc-tetrofosmin SPECT-MPI for evaluation of known or suspected coronary artery disease (CAD) between May 2005 and December 2008. Patients were followed for a median of 2.7 years for CAD-related events and the occurrence of MACE (unstable angina, coronary artery revascularization, non-fatal myocardial infarction, or death). The study protocol was approved by the local ethics committee (BASEC No. 2017-01112).

### Myocardial perfusion imaging by ^99m^Tc-tetrofosmin SPECT

Patients underwent electrocardiography (ECG)–gated 1-day stress/rest ^99m^Tc-tetrofosmine SPECT-MPI on a dual-head camera (Infinia Hawkeye or Ventri, both GE Healthcare, Milwaukee, WI, USA) [3]. Cedars QGS/QPS software (Cedars-Sinai Medical Center, Los Angeles, CA, USA) was used for SPECT-MPI semi-quantitative image analysis according to current guidelines. Attenuation correction and coronary calcium scoring (CACS) were obtained from non-contrast computed tomography (CT) using a 64-slice CT scanner (LightSpeed VCT, GE Healthcare).

### CCTA acquisition and fat attenuation index measurements

**Fig. 1:**
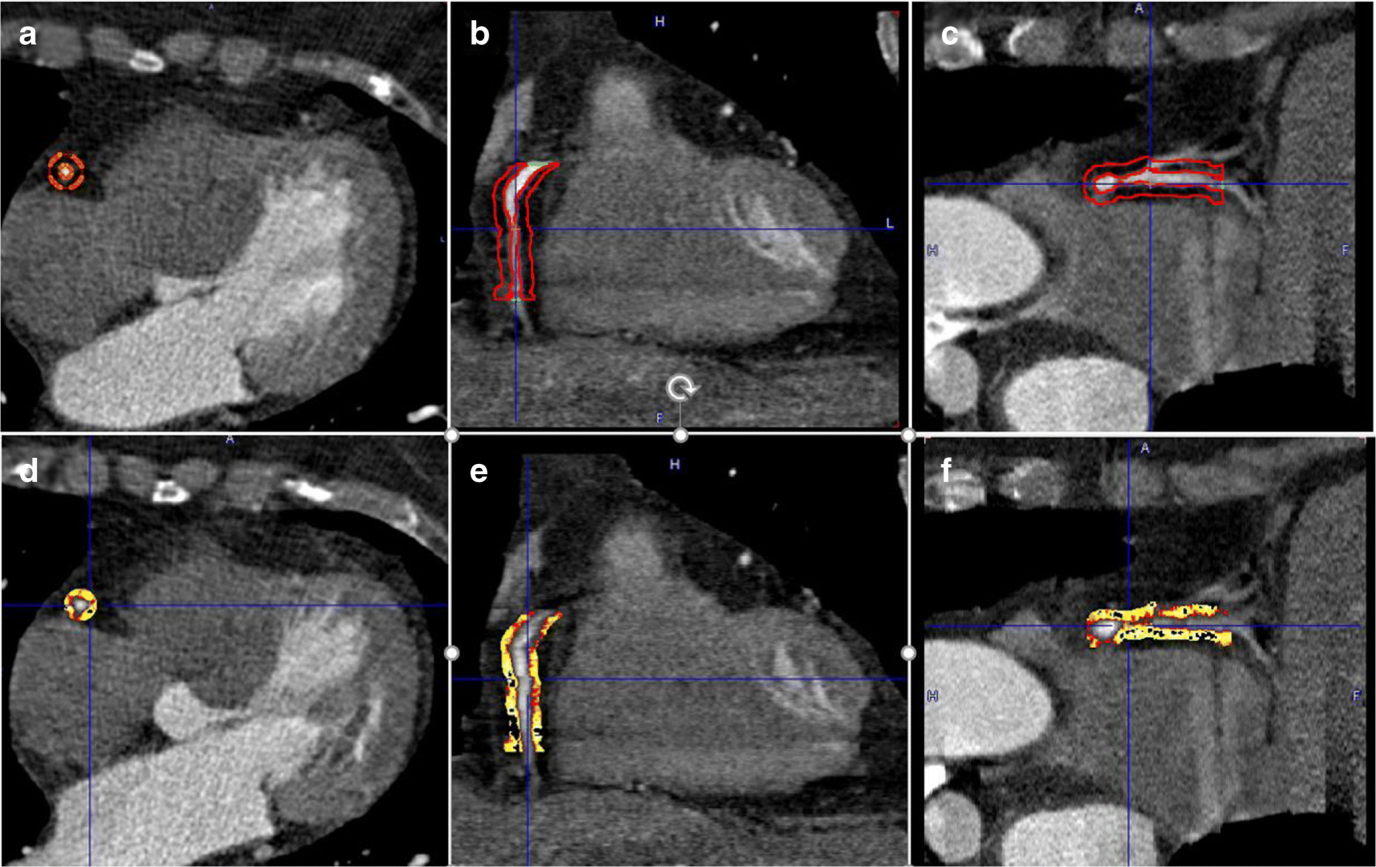
Perivascular fat attenuation index (FAI) phenotyping from coronary computed tomography angiography (CCTA) in the proximal 10–50 mm of the right coronary artery (RCA). **a**–**c** Volume of interest (VOI) in transaxial, coronal, and sagittal views. **d**–**f** FAI phenotyping in transaxial, coronal, and sagittal views

CCTA scans in this study were acquired on a stand-alone 64 slice CT LightSpeed VCT scanner (GE Healthcare). About 15% of CCTA scans were performed on a Siemens Somatom scanner (Siemens Healthineers), using a contrast-enhanced method with helical scanning. FAI measurements were carried out by a semi-automated analysis using PMOD V 3.805 (PMOD Technologies, Zurich, Switzerland). The right coronary artery (RCA), the left anterior descending artery (LAD), and the left main coronary artery (LMCA) were traced for approximately 50 mm starting at their origin. To avoid artifacts of adjacent vessels (e.g., aorta to RCA), the ostial 10 mm of the RCA and LMCA and the ostial 10 mm of the LAD were excluded. Hence, the proximal 10–50 mm of the RCA (Fig. 1) and LAD and the available proximal length of the LMCA were analyzed. Upon identifying the relevant vessel segments, PVAT was defined as the surrounding adipose tissue within a radial distance from the outer artery wall equal to the diameter of the respective artery, as previously reported [1, 2]. Within these tube-shaped volumes of interest, the adipose tissue was defined as all voxels ranging from − 190 to − 30 Hounsfield units (HU). While the observer was required to place markers in the respective vessel to determine the length to be analyzed, the cylindrical starting volume and segmentation according to HU range was controlled by the software. FAI was calculated as the average HU over all voxels defined as PVAT.

### Statistical analysis

Continuous variables are presented as mean ± standard deviation (SD) and categorical variables as frequency or percentage. The Mann-Whitney *U* test, Student’s *t* test, analysis of variance (ANOVA), or Kruskal-Wallis test were used for group comparisons of continuous variables. Cumulative event-free survival curves for MACE were compared across categories of FAI by using the Kaplan-Meier analysis and log-rank test. The effect size of FAI categories on MACE was analyzed with a Cox proportional hazards survival model. A two-tailed *p* value of ≤ 0.05 was considered statistically significant. All statistical analyses were performed with SPSS (SPSS Statistics for Windows Version 24.0., IBM Corp., Armonk, NY).

## Results

###  FAI and coronary calcium score

Baseline characteristics of all 492 patients (173 women, 35%) stratified by sex are depicted in Table 1. Perivascular FAI was measured around the RCA, the LMCA, and the LAD (Table 1, FAI[RCA], FAI[LMCA], and FAI[LAD], respectively). While FAI[RCA] and FAI[LAD] were higher in men than in women (− 77.8 ± 9.7 vs − 80.5 ± 10.4, *p* = 0.009; and − 79.4 ± 8.8 vs − 81.4 ± 8.7, *p* = 0.024), there was no significant sex difference in FAI[LMCA] values, as indicated in Table 1 (73.9 ± 11.6 vs − 74.9 ± 13.7, *p* = 0.58). There was no significant correlation between CACS and FAI in any coronary artery, independent of sex (data not shown).

**Table 1:** Patient baseline characteristics and cardiovascular imaging parameters

Patient baseline characteristics	Overall population *n* = 492	Men *n* = 319	Women *n* = 173	*p* value (2-sided)
Age (years), mean ± SD	62.52 ± 10.75	61.38 ± 10.54	64.62 ± 10.86	0.001
BMI (kg/m^2^), mean ± SD	26.86 ± 4.60	27.03 ± 4.15	26.56 ± 5.33	0.277
Diabetes, *n* (%)	70 (14.3)	55 (17.3)	15 (8.7)	0.010
Hypertension, *n* (%)	302 (61.6)	179 (56.3)	123 (71.5)	0.001
Smoking, *n* (%)	166 (33.9)	121 (38.1)	45 (26.2)	0.009
Hypercholesterolemia, *n* (%)	238 (48.6)	156 (49.1)	82 (47.7)	0.777
Obesity, *n* (%)	96 (19.5)	54 (16.9)	42 (24.3)	0.057
Family history of CAD, *n* (%)	169 (34.5)	99 (31.1)	70 (40.7)	0.037
Known CAD, *n* (%)	98 (20.0)	80 (25.2)	18 (10.5)	< 0.001
Previous MI, *n* (%)	38 (7.8)	29 (9.1)	9 (5.2)	0.157
Previous PCI, *n* (%)	69 (14.1)	51 (16.0)	18 (10.5)	0.103
Previous CABG, *n* (%)	18 (3.7)	18 (5.7)	0 (0.0)	0.001
ACE inhibitor, *n* (%)	108 (45.0)	72 (44.7)	36 (45.6)	1.000
Beta-blocker, *n* (%)	118 (49.2)	77 (47.8)	41 (51.9)	0.584
Aspirin, *n* (%)	161 (67.1)	110 (68.3)	51 (64.6)	0.562
Statin, *n* (%)	91 (37.9)	67 (41.6)	24 (30.4)	0.119
CACS (AU), mean ± SD	450.30 ± 785.93	553.12 ± 857.61	246.78 ± 571.53	0.002
LVEF (%), mean ± SD	63.13 ± 11.77	60.13 ± 11.61	68.95 ± 9.75	< 0.001
LVEF ≥ 50%, *n* (%)	320 (88.4)	202 (84.5)	118 (95.9)	0.001
Abnormal MPI, *n* (%)	103 (24.9)	82 (29.7)	21 (15.2)	0.001
Ischemia, *n* (%)	75 (18.1)	58 (21.0)	17 (12.3)	0.031
Stenosis, *n* (%)	153 (40.8)	114 (46.7)	39 (29.8)	0.001
MACE, *n* (%)	56 (17.8)	41 (19.2)	15 (15.0)	0.430
FAI[RCA] (HU), mean ± SD	− 78.76 ± 10.06	− 77.77 ± 9.73	− 80.45 ± 10.43	0.009
FAI[LCMA] (HU), mean ± SD	− 74.19 ± 12.25	− 73.87 ± 11.57	− 74.89 ± 13.66	0.584
FAI[LAD] (HU), mean ± SD	− 80.11 ± 8.76	− 79.38 ± 8.76	− 81.38 ± 8.65	0.024
IFV (cm^3^), mean ± SD	125.06 ± 54.32	129.51 ± 49.89	115.89 ± 61.73	0.041
EFV (cm^3^), mean ± SD	258.42 ± 106.90	277.16 ± 100.74	219.80 ± 109.35	< 0.001

Characteristics of the study population stratified by sex. Data are presented as mean ± SD and *n* (%). Two-sided *p* values are indicated

*ACE*, angiotensin-converting enzyme inhibitor; *BMI*, body mass index; *CABG*, coronary artery bypass grafting; *CACS*, coronary artery calcification score; *CAD*, coronary artery disease; *EFV*, epicardial fat volumes; *FAI*, fat attenuation index; *IFV*, intrathoracic fat volumes; *LAD*, left anterior descending artery; *LCMA*, left coronary main artery; *LVEF*, left ventricular ejection fraction; *MACE*, major adverse cardiovascular events; *MI*, myocardial infarction; *MPI*, myocardial perfusion imaging; *PCI*, percutaneous coronary intervention; *RCA*, right coronary artery; *SD*, standard deviation

### Clinical endpoints according to FAI

**Fig. 2:**
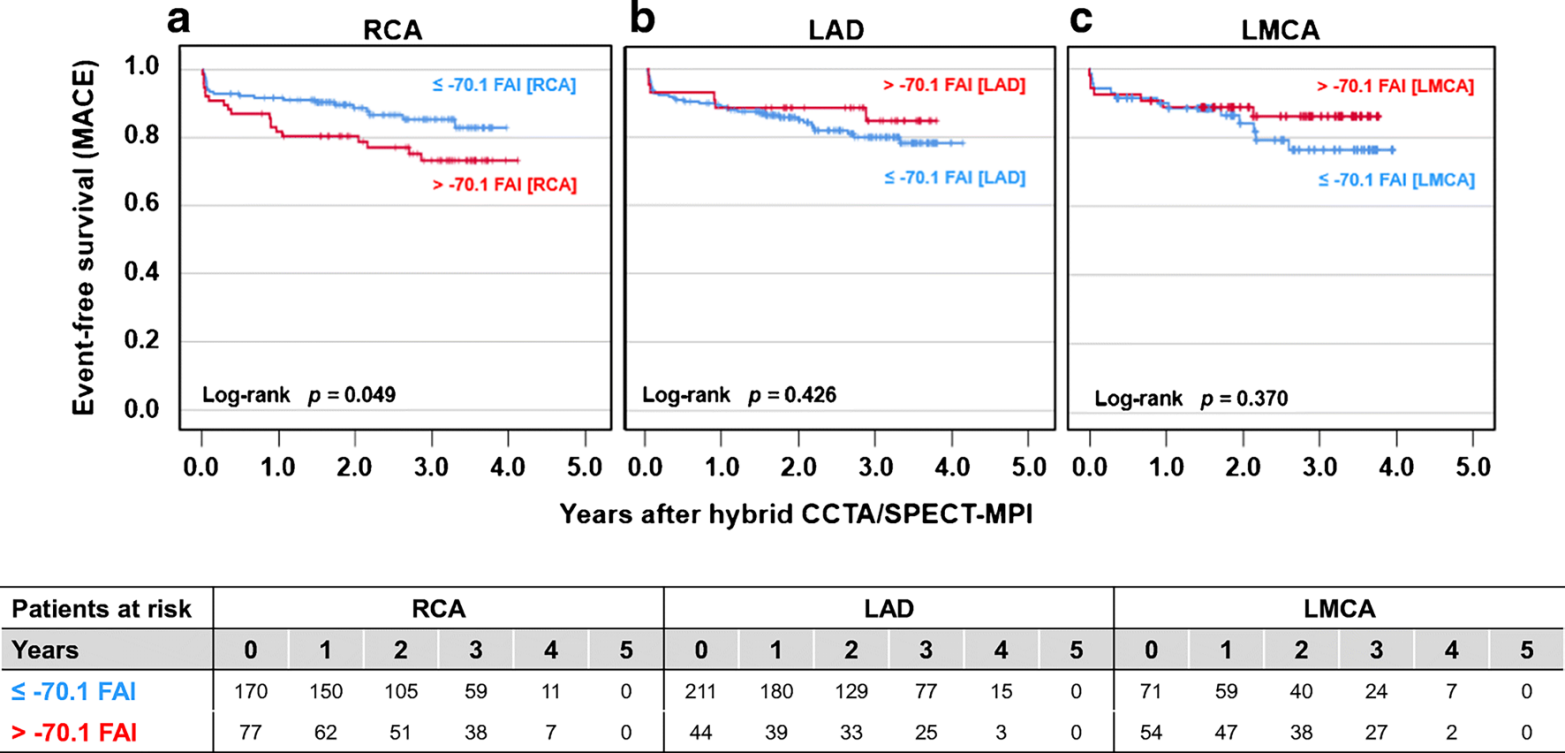
**a** Kaplan-Meier survival curves for the occurrence of major adverse cardiac events (MACE) in patients with low (≤ − 70.1 HU) versus high (> − 70.1 HU) FAI[RCA]. **b** Kaplan-Meier survival curves for the occurrence of major adverse cardiac events (MACE) in patients with low (≤ − 70.1 HU) versus high (> − 70.1 HU) FAI[LAD]. **c** Kaplan-Meier survival curves for the occurrence of MACE in patients with low (≤ − 70.1 HU) versus high (> − 70.1 HU) FAI[LMCA]. *FAI*, fat attenuation index; *RCA*, right coronary artery; *LAD*, left anterior descending artery; *LCMA*, left coronary main artery

In a subcohort of 314 patients (100 women, 32%), information on clinical endpoints was obtained over a median follow-up time of 2.7 years. Among these individuals, 56 patients experienced at least one cardiac event (i.e., cardiac death, non-fatal myocardial infarction, hospitalization for any cardiac reason, and late revascularization during follow-up). Increased FAI[RCA] > − 70.1 was associated with an increased rate of MACE during follow-up (log-rank *p* = 0.049; Fig. 2a), while FAI[LAD] and FAI[LMCA] were not associated with clinical endpoints (log-rank *p* = 0.426 and 0.370, respectively; Fig. 2b and c). Similar trends and results were observed when patients with obstructive CAD were excluded from the analysis (FAI[RCA] log-rank *p* = 0.015, FAI[LAD] log-rank *p* = 0.367, and FAI[LMCA] log-rank *p* = 0.148, respectively).

### Predictors of MACE

A multivariate Cox regression model taking into account age, sex, BMI, cardiovascular risk factors, and obstructive CAD (> 50% luminal narrowing on CCTA) identified FAI[RCA] as an independent predictor of MACE (HR 2.874, 95% CI: 1.280–6.450, *p* = 0.010; Table 2 part A). When reversible perfusion defect from MPI was added as a covariate, FAI[RCA] remained an independent and significant predictor of MACE (HR 2.733, 95% CI: 1.220–6.123, *p* = 0.015, Table 2 part B). However, when CACS results were included in the model, FAI[RCA] did no longer offer incremental prognostic value for the prediction of MACE (*p* = 0.279; Table 2 part C). A first-order interaction term consisting of sex and FAI[RCA] was significant in all regression models (Table 2 part A–C, HR 2.151, 95% CI: 1.252–3.697, *p* = 0.006; HR 2.119, 95% CI: 1.218–3.686, *p* = 0.008; and HR 2.071, 95% CI: 1.111–3.861, *p* = 0.022). FAI[LAD] and FAI[LMCA] were not selected as significant predictors of MACE by any of the models (data not shown).

**Table 2:** Multivariate regression analysis for the prediction of major adverse cardiovascular events (MACE)

Independent variables	OR (95% CI)	*p* value
A. Multivariate Cox regression model for the prediction of MACE, adjusted for cardiovascular risk factors and obstructive CAD on CCTA		
BMI	1.127 (1.050–1.209)	0.001
Obstructive CAD	4.980 (2.132–11.630)	< 0.001
FAI[RCA] > − 70.1	2.874 (1.280–6.450)	0.010
B. Multivariate Cox regression model for the prediction of MACE, adjusted for cardiovascular risk factors, obstructive CAD on CCTA, and reversible perfusion defect by SPECT-MPI		
BMI	1.106 (1.026–1.192)	0.008
Hypercholesterolemia	2.349 (0.945–5.837)	0.066
Obstructive CAD	4.154 (1.759–9.809)	0.001
FAI[RCA] > − 70.1	2.733 (1.220–6.123)	0.015
C. Multivariate Cox regression model for the prediction of MACE, adjusted for cardiovascular risk factors, obstructive CAD on CCTA, and reversible perfusion defect by SPECT-MPI and CACS		
Hypercholesterolemia	2.790 (1.073–7.255)	0.035
Obstructive CAD	3.360 (1.137–9.926)	0.028
Reversible perfusion defect	2.478 (0.934–6.573)	0.068

A–C, the stepwise method was performed among age, body mass index (BMI), sex, previous percutaneous coronary intervention (PCI) or coronary artery bypass graft (CABG), diabetes mellitus, hypercholesterolemia, hypertension, family history of CAD, current smoking, and fat attenuation index of the right coronary artery (FAI[RCA] > − 70.1). Only variables staying in the final model are presented

*CACS*, coronary artery calcium score; *CAD*, coronary artery disease; *CI*, confidence interval; *OR*, odds ratio; *SPECT*, single-photon emission computed tomography; *MPI*, myocardial perfusion imaging; *CCTA*, coronary computed tomography angiography; *RCA*, right coronary artery

## Discussion

Our study is the first to report the prognostic value of perivascular FAI in patients undergoing both CCTA and SPECT-MPI. Our findings indicate that quantifying perivascular FAI does not add incremental prognostic value beyond multimodality MPI/CCTA findings including CACS. A significant interaction between sex and FAI observed in our study emphasizes the need to take sex differences in the diagnostic accuracy of FAI into account.

Consistent with previous reports, perivascular FAI was associated with adverse cardiovascular events in unadjusted analyses [1, 2]. However, FAI no longer offered information gain when CACS was available. The strong link between arterial calcification and inflammation might account for this observation. Indeed, studies have demonstrated that macrophages are implicated in the osteogenic transition of vascular smooth muscle cell (VSMC), thereby triggering the release of VSMC- and macrophage-derived matrix vesicles—precursors of microcalcification [4]. Further, Aikawa et al. suggested that the real-time assessment of macrophage burden is directly linked to osteogenic activity in early-stage atherosclerosis [5]. Of note, however, we did not observe a significant correlation between CACS and FAI in our study population. The latter might be due to the fact that inflammation becomes less prominent during the final stages of macroscopic calcification [6]. Accordingly, studies of the association between high-sensitivity C-reactive protein (hs-CRP) and CACS in healthy subjects have yielded largely conflicting results [7, 8]. Thus, future studies will have to elucidate whether CACS and FAI may provide independent or complementary information regarding the risk of cardiovascular events.

While increased FAI (≥ − 70.1) in the RCA was associated with an increase in MACE in unadjusted analyses, no such association was found for the LAD or LCMA in our study. High interindividual variability in LMCA length with a distinct inherent distribution pattern of PVAT in different individuals may have accounted for these findings. While previous studies do not report data on LMCA FAI, Oikonomou et al. demonstrated an association between an enhanced FAI in the LAD and increased MACE in their study which contrasts with our report [2]. Differences in inclusion criteria, study endpoints, and the fact that the prognostic value of FAI seems to depend on the presence and localization of culprit lesions may have accounted for the divergent findings. In addition, the cut-off point of − 70.1 has only been validated for FAI[RCA] but not for other regions by Oikonomou et al. [2]. Therefore, the definition of an optimal cut-off for other coronary segments including the LAD or LMCA warrants further study.

In our study, FAI was significantly higher in men than in women. In addition, a significant interaction between FAI and sex with regard to cardiovascular outcomes was observed in our study indicating that sex differences impact the prognostic value of FAI. Given that sex differences in the association between inflammation and CAD are well described, higher baseline concentrations of circulating inflammatory markers in women might impact perivascular adipogenesis irrespective of the presence or absence of high-risk coronary plaque features [9]. Further, microvascular dysfunction is more common in women than in men [10]; thus, quantification of vascular inflammation in the epicardial arteries may not reliably predict adverse cardiovascular outcomes that result from microvascular disease in women. Further research is needed to identify sex-specific variables that allow the accurate interpretation of atherosclerotic high-risk features in women with CAD.

There are limitations to this study that should be pointed out. First, this study is a single-center retrospective analysis conducted in a cohort with high prevalence of CAD and comorbidities, which limits its generalizability. Second, although a comprehensive group of adjustment variables was employed, unmeasured factors such as the influence of obesity on FAI measurements may have affected our endpoints. As such, our study did not account for the initiation of aspirin and/or statin treatment after the initial CCTA, which may have interfered with the predictive value of FAI for long-term mortality, as previously suggested [2]. Third, the binary cut-off value for FAI in our study was chosen based on previous reports [1] as currently no standardized quantitative categories for FAI are available. Finally, the follow-up period of our study was relatively short, and an extended follow-up might have provided more complete information on the predictive value of FAI.

In summary, we demonstrate that FAI does not offer information gain when CACS is available. Given the widespread use of routine CCTA and the implications of vascular inflammation in a variety of disease conditions, further studies are warranted to enable optimal use of FAI in cardiovascular risk stratification, to compare the clinical utility of FAI with other emerging biomarkers such as CT-derived fractional flow reserve (CT_FFR_), and to explore alternative modalities for PVAT assessment including [^18^F]FDG-positron emission tomography (PET).
